# The Gap between Tobacco Treatment Guidelines, Health Service Organization, and Clinical Practice in Comprehensive Cancer Centres

**DOI:** 10.1155/2011/145617

**Published:** 2011-07-07

**Authors:** R. Mazza, M. Lina, G. Invernizzi, M. Pierotti, C. De Marco, C. Borreani, R. Boffi

**Affiliations:** ^1^Patient Information Services, Fondazione IRCCS Istituto Nazionale dei Tumori, 20133 Milan, Italy; ^2^Tobacco Control Unit, Fondazione IRCCS Istituto Nazionale dei Tumori, 20133 Milan, Italy; ^3^Psychology Unit, Fondazione IRCCS Istituto Nazionale dei Tumori, 20133 Milan, Italy; ^4^Società Italiana di Medicina Generale, 50142 Florence, Italy; ^5^Scientific Direction, Fondazione IRCCS Istituto Nazionale dei Tumori, 20133 Milan, Italy; ^6^Tobacco Control Unit, Department of Medical Oncology, Fondazione IRCCS Istituto Nazionale dei Tumori, 20133 Milan, Italy

## Abstract

Smoking cessation is necessary to reach a higher quality of life, and, for a cancer patient, it represents an important step in improving the outcome of both prognosis and therapy. Being a cancer patient addicted to nicotine may be a critical situation. We conducted a survey to monitor how many comprehensive cancer centres in Italy have an outpatient smoker clinic and which kinds of resources are available. We also inquired about inpatient services offering psychological and pharmacological support for smoking cessation, reduction, or care of acute nicotine withdrawal symptoms. What we have witnessed is a significant gap between guidelines and services. Oncologists and cancer nurses are overscheduled, with insufficient time to engage in discussion on a problem that they do not consider directly related to cancer treatment. Furthermore, smoking habits and limited training in tobacco dependence and treatment act as an important barrier and lead to the undervaluation of smokers' needs.

## 1. Introduction

“It is a journey, not a fact”: this redefinition of smoking cessation by Swartz Woods and Jaen [1] is particularly significant for smokers with a medical diagnosis. In this approach, every exchange between health system and users may have an educative impact, and not only on the patient. Hospitalization is a “teachable moment” [2] as is every encounter with health personnel an opportunity to interfere with the tobacco epidemic, the most preventable of the world's health problems and responsible for killing more people than AIDS, tuberculosis, and malaria put together [3]. In many illnesses, treating tobacco use and dependence becomes part of the patient's care. This is the case for the treatment of tobacco-related diseases, such as in

- most cardiovascular treatments [4],
- cancer care [5],
- respiratory treatments [6],
- every surgical treatment [7],
- treatment of patients who need inhaled drugs [8],
- prevention of immune system impairment [9, 10].

These considerations should lead to the implementation of a sound policy in every health organization, but the gap between theory and clinical practice becomes increasingly obvious. 

In the last few years, Italy has made political progress in the field of tobacco control, such as the 2005 law ensuring smoke-free workplaces and hospitality venues. However, 11.1 million (21.7%) adults in Italy are still current smokers [11], and only 375 accredited antismoking centres are operating [12].

Whereas the law is being respected in almost all bars and restaurants, irony wants it that many hospitals are still lagging behind in its implementation. We surveyed the major general hospitals in Milan, the heart of Lombardy's Health System and one of the most developed regions in Italy. Not one of the eight Milan General Hospitals made the NRT (nicotine replacement therapy) available to clinicians to deal with smoking cessation or acute nicotine withdrawal syndrome developed during the inhospital stay. In October 2008, we attended a national Health Promoting Hospitals' Conference, and were impressed by what was stated on the situation of tobacco control in three of the eight Milan General Hospitals. In the first hospital, “the No-Smoking Ban is frequently eluded not only in places like coffee break areas, bathrooms, locker rooms, offices, but also in places frequented by patients like examination rooms, the infirmary and recovery rooms.” This study was signed by the Medical and Surveillance Services and by the Quality Control Service. In the second hospital, the study conducted by the Pneumology Rehabilitation Services showed that 45% of nonsmoker employees are exposed to second-hand smoke during working hours. In the third general hospital, another study signed by the Pneumology Medical Board indicated that non-smokers exposed to second-hand smoke in hospitals accounted for 83% for men and 88.4% for women [13].

The cancer “setting” might be a good chance to implement the smoking cessation guidelines, considering that 23.9% of all cancer deaths (33.4% men and 9.6% women) is due to smoking habits [14].

Among hospitalized cancer patients, smokers constitute about 24.5% of the total figure and former smokers about 48.2% [15].

Smoking cessation is an important part of cancer treatment. Different studies indicate that the smoking habit influences the outcome of surgical intervention [16], chemotherapy [17, 18], radiotherapy [19], and biological therapies [20].

Smoking cessation is also part of the treatment in the following conditions: lung cancer [21], breast reconstruction using the free TRAM flap [22], liver transplant [23, 24], colorectal surgery [25], and bone marrow transplant [26].

Evidence-based treatments of smoking habit in oncological patients include nicotine replacement therapies, bupropion, varenicline, and behavioural counselling provided individually, in groups, or by telephone [27–29]. However, clinical practice in Italian cancer departments is still far from such a standard. 

In the year 2000, at the National Cancer Institute of Milan, we initiated a smoke-free campaign addressed to patients, visitors, and health personnel. The Institute became a member of the network of smoke-free hospitals, and, since 2003, an antismoking centre has been operating with an average of 350 smokers treated each year. We are at present providing pharmacological and psychological support as an inpatient service to take care of hospitalized smokers. Our pharmacy offers nicotine replacement therapy (NRT), bupropion, and varenicline for smoking cessation and treatment of inpatients' severe acute nicotine withdrawal syndrome. We have summarized, in Table 1, the acute nicotine withdrawal symptoms. Differential diagnosis of this syndrome is made by trained ward clinicians or by neurologists who call upon the intervention of the inpatient antismoking service. In Table 2, we report the last 11 smoker patients diagnosed with this syndrome and treated with NRT. This kind of therapy usually resolves patients' symptoms within 24 hours. 

However, our services are merely a drop in the ocean: Italy has 11 million smokers, and a considerable part of the population (approximately 4% or 2.250.000 people, in 2006) lives with a prior cancer diagnosis [30].

We carried out a telephone survey to study the Italian offer of treatment against tobacco use and dependence in 17 cancer centres (CC) belonging to “Alleanza contro il Cancro” (a government initiative that creates a network amongst the most important cancer institutes or cancer departments in general hospitals). 

We focussed our survey on the services provided to smoker outpatients (with or without an oncological illness) and on the services provided for inpatients with lung or head and neck cancer. Our first step was to make a telephonic enquiry at the different Institution desks, asking for an outpatient antismoking clinic, and we then contacted the clinic personnel.

In order to favour the cessation or reduction of smoking, six of these clinics offer both psychological and pharmacological support while one only offers group therapy. In Tables 3, 4, and 5, we describe the organization and the resources of the 7 existing outpatient Clinics.

For the second part of the survey, we contacted the head nurses of the 12 lung cancer wards and of the 11 head and neck cancer wards. 

NRT, bupropion, and varenicline are available for inpatients in only one cancer clinic (Table 5). Among the existing 12 lung cancer wards only one offers psychological and pharmacological support for patients motivated or compelled to quit smoking in order to undergo treatment and specific surgery. Acute withdrawal syndrome is usually not detected nor treated and not bedridden cancer inpatients continue to smoke during treatment, including the days prior and following surgery, with the exception of three wards (Table 6). In these cases head nurses, able to make a differential diagnosis, remembered the presence of an acute syndrome among their patients. However, only one ward had a specific inpatient service to deal with such a situation. In one ward, patients have been administered NRT drugs bought by relatives in pharmacies outside the hospital while in another ward patients are treated by anaesthetists without using NRT/bupropion/varenicline.

We observed the same situation in the existing eleven head and neck cancer wards. Only one ward provides an inpatient tobacco-use treatment service and a comprehensive acute nicotine withdrawal syndrome care. The second ward, listed in the table, treats the syndrome with NRT drugs bought by relatives outside the hospital (Table 7).

## 2. Conclusions

Being a cancer patient addicted to nicotine may be a critical situation. Oncologists and cancer nurses are overscheduled, with insufficient time to engage in discussion on a problem that they do not consider directly related to cancer treatment. Health personnel's smoking habits [31] and limited training in tobacco dependence and treatment act as an important barrier to progress and lead to the undervaluation of smokers' needs. 

What must be recognized is that this kind of care can be a great opportunity, not only to support, but to empower smoker cancer patients and to motivate profound changes in their lifestyle. In the cases of acute nicotine withdrawal symptoms, assistance can be of great comfort and help and can further improve relations between patient and hospital operators. It also works towards permanent results in smoking cessation and increases compliance with hospital no-smoking policies [29, 32]. 

It is of crucial importance that oncologists of every comprehensive cancer centre offer patients, and the whole community, a service of smoking cessation; this service should be connected with the national quit line and with the antismoking centres network. They should also advocate for tobacco-free environments in their patients' communities and share, with their colleagues, their experience in dealing with the tobacco epidemic [33].

The basic needs of smoker cancer patients affect human rights, and recognizing this is an essential part in creating a quality approach to cancer care.

With a nonjudgmental and relationship-centred approach, the aim of the intervention should be to inform and support smoker cancer patients to identify and reach their own health goals according to their own needs and resources.

##  Conflict of Interests

The authors declare that there is no conflicts of interest.

## Figures and Tables

**Table 1:** According to Hughes J. R. Nicotine withdrawal symptoms. Effects of abstinence from tobacco: valid symptoms and time course.

Withdrawal Symptoms	Peak	Duration
Anger/irritability/frustration	Within first week	2, 4 or more weeks
Anxiety	3 day	2 weeks
Dysphoria (depressed mood and negative affect)	1, 2, 3 weeks	4 weeks
Difficulty concentrating	2, 3 days	3, 4 weeks
Impatience		3, 4, or more weeks
Insomnia (sleep fragmentation)		
Restlessness	1, 3 days	2, 4 weeks

**Table 2:** Characteristics of the last 11 inpatients diagnosed with acute withdrawal symptoms and treated at the National Cancer Institute of Milan.

Gender	Age	Disease	Setting	Symptoms	Therapy
M	56	Bladder cancer	Surgery	Restlessness	NRT inhaler
F	42	Breast cancer	Reconstructive	Dysphoria, insomnia	NRT inhaler
M	34	Metastatic sarcoma	Palliative	Anxiety, restlessness, insomnia	NRT patch
F	74	Oropharyngeal cancer	Surgery	Restlessness	NRT patch
F	43	Metastatic ovary cancer	Surgery	Anxiety, insomnia, difficulty concentrating	NRT patch, inhaler
M	66	Lung cancer	Surgery	Anxiety, restlessness	NRT patch
F	52	Metastatic breast cancer	Chemotherapy	Insomnia, restlessness, dysphoria	NRT patch, inhaler
M	56	Oropharyngeal cancer	Surgery	Restlessness, craving	NRT patch
M	28	Kidney cancer	Palliative	Anxiety, insomnia, craving	NRT patch, inhaler
M	28	Nose cancer	Palliative	Restlessness, craving	NRT patch, inhaler
M	62	Colon cancer	Surgery	Anxiety, restlessness, insomnia	NRT patch

**Table 3:** Outpatient clinics' treatments for tobacco use and dependence.

	Yes (%)	No (%)
NHS-funded antismoking clinic	5 (29.41)	12 (70.59)
Private antismoking clinic	2 (11.76)	15 (88.24)

**Table 4:** Outpatient Clinics' multidisciplinary team.

	Yes (%)	No (%)
Physician	6 (85.71)	1 (14.29)
Psychologist	7	0
Nurse	2 (28.57)	5 (71.43)
Nutritionist	1 (14.29)	6 (85.71)
Pulmonary physiotherapist	1 (14.29)	6 (85.71)

**Table 5:** Pharmacological treatments at surgical wards disposal to support inpatient smoking cessation or to care acute nicotine withdrawal syndrome in the 17 cancer centres.

	Yes (%)	No (%)
Availability of NRT, bupropion, and/or vareniclina at Hospitals' pharmacy	1 (5.88)	16 (94.12)

**Table 6:** Treatments of tobacco use and dependence for lung cancer inpatients in the existing 12 surgical wards.

	Yes (%)	No (%)
Smoking cessation: pharmacological support	1 (8.33)	11 (91.67)
Smoking cessation: psychological support	1 (8.33)	11 (91.67)
Acute nicotine withdrawal syndrome care	3 (25)	9 (75)

**Table 7:** Treatments of tobacco use and dependence for head and neck cancer inpatients in the existing 11 surgical wards.

	Yes (%)	No (%)
Pharmacological support	1 (9.09)	10 (90.91)
Psychological support	1 (9.09)	10 (90.91)
Acute nicotine withdrawal syndrome care	2 (18.18)	9 (81.82)
